# Supplementary material for: Uncooled sub-GHz spin bolometer driven by auto-oscillation
Source: Nat Commun. 2021 Jan 26;12:536. doi: 10.1038/s41467-020-20631-0 (PMC7838188; doi:10.1038/s41467-020-20631-0)
Supplement: Supplementary file 1 — Supplementary Information [file 41467_2020_20631_MOESM1_ESM.pdf]

Supplementary Information

Uncooled sub-GHz Spin Bolometer Driven by Auto-oscillation

Minori Goto, Yuma Yamada, Atsushi Shimura, Tsuyoshi Suzuki,

Naomichi Degawa, Takekazu Yamane, Susumu Aoki, Junichiro Urabe,

Shinji Hara, Hikaru Nomura and Yoshishige Suzuki

Supplementary Note 1: Mechanism of the proposed spin bolometer

We attribute the high diode voltage to the nonlinear diode effect[1] due to the heat-induced spin-torque under spin-torque auto-oscillation conditions. Nonlinear diode effects are generated by magnetization precession with a nonlinear magnetization potential. Under such a potential, the precession center depends on the magnitude of the precession angle, which induces a dc resistance change ΔR. The dc bias current I_{dc} and resistance change ΔR generate a nonlinear diode voltage $(\Delta R)I_{dc}$. Large spin-torque diode responsivities are attributed mainly to nonlinear diode effects[1]. Moreover, in our system, the resistance change is induced by spin-torque auto-oscillation[2] and HCMA[3]. Spin-torque auto-oscillation is excited by the anti-damping torque due to the dc bias current[2]. A previous study reported a high spin-torque diode using

spin-torque auto-oscillation[4]. Under our experimental conditions, the input microwave synchronizes through the HCMA with the magnetization precession of spin-torque auto-oscillation and generates the diode voltage. As reported in a previous study[3], HCMA in MgO|FeB|MgO systems exerts spin-torque on magnetization more effectively than the spin-transfer torque and voltage-controlled magnetic anisotropy. In our study, the spin-torque auto-oscillation synchronizes with the spin-torque induced by the HCMA effect to generate a high diode voltage.

Supplementary Note 2: Measurement circuit and device characteristics

The circuit used for the spin-torque diode measurements is shown in Fig. S1(a). Microwaves were applied to the magnetic tunnel junction (MTJ) from a signal generator. The diode voltage was measured by a lock-in amplifier synchronized with the signal generator. We used an attenuator of −50 dB to reduce the intensity of microwaves. A magnetic field was applied with intensity B, azimuthal angle θ, and rotation angle φ. Figures S1(b) and S1(c) show the in-plane and out-of-plane magnetic field dependence of the resistance of the MTJ, respectively. The in-plane magnetic field is applied in the $+y$ direction, which is opposite to that of the pinned layer magnetization. The solid and dashed lines represent the sweep direction of the magnetic field. We obtained a magneto-resistance ratio of 43%, and a resistance-area product of 3.9 $\Omega\mu m^2$.

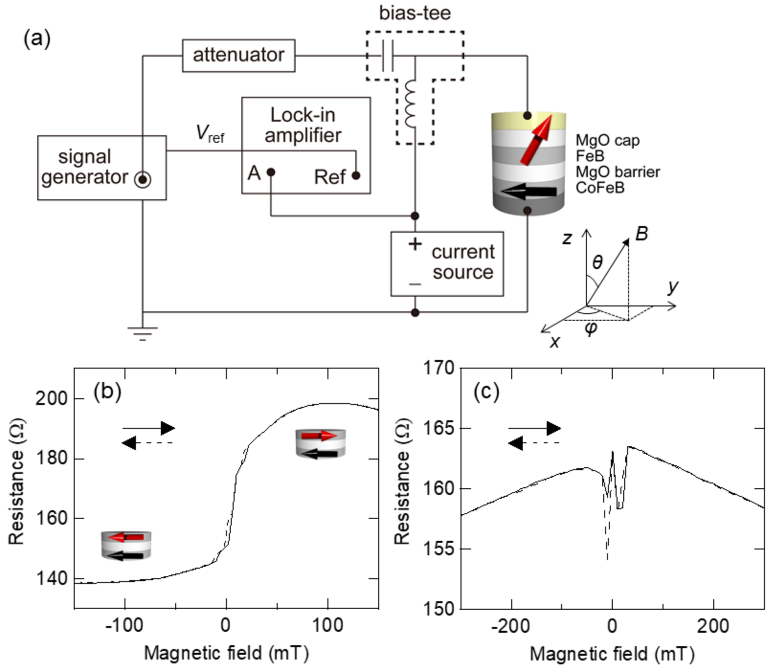

Supplementary Figure S1.(a) Schematic of spin-torque diode measurement circuit. Microwaves with amplitude modulation frequency of 7.5 kHz are applied to the MTJ from a signal generator synchronized with a lock-in amplifier. Bold red and black arrows represent free and pinned layer magnetization, respectively. The magnetic field is applied with intensity *B*, azimuthal angle *θ,* and rotation angle *φ*. (b) In-plane and (c) out-of-plane magnetic field dependence of the MTJ resistance. The in-plane magnetic field is applied in the +*y* direction. The solid and dashed lines represent the sweep direction of the magnetic field.

Supplementary Note 3: Elevation angle dependence of diode voltage

We measured the dependence of the diode voltage on the elevation angle of the magnetic field to confirm the existence of the nonlinear diode voltage. The measurement circuit is the same as that shown in Fig. S1(a), and the attenuation is set to −30 dB. The amplitude modulation frequency is 7.5 kHz. The film structure of the MTJ is the same as that described in the main text, and the diameter is 130 nm. A magnetic field of 100 mT is applied along the *y*–*z* plane as shown in Fig S2(a). Under this condition, the magnetization in the FeB free-layer is almost saturated. The elevation angle of the magnetic field, θ_H, is defined as shown in Fig. S2(a). The dc current is approximately −1.39 mA.

Figure S2(b) shows the dependence of the diode voltage on θ_H. The sign of the diode voltage is positive in the range $-90° < \theta_H < 90°$ and negative in the range $90° < \theta_H < 270°$. This result is consistent with the symmetry of the nonlinear diode voltage under the negative bias current applied to the in-plane magnetized MTJ as discussed below.

The energy density of magnetization under perpendicular magnetic anisotropy and an external magnetic field is described by

$$E = -K_z \sin^2\theta_m - \mu_0 M_s H \cos\left(\theta_m - \theta_H\right). \quad \text{(S1)}$$

Here, K_z is the perpendicular magnetic anisotropy, M_s is the saturation magnetization, H is the external magnetic field, and θ_m is the elevation angle of magnetization as shown in Fig. S2(a). We define the slight difference between the magnetization and the magnetic field as $\Delta\theta = \theta_m - \theta_H$. Eq. (S1) is expanded in terms of $\Delta\theta$ as follows:

$$\begin{aligned} E = &-\left(K_z \sin^2\theta_H + \mu_0 M_s H\right) - K_z \sin 2\theta_H \,\Delta\theta - \left(K_z \cos 2\theta_H - \frac{1}{2}\mu_0 M_s H\right)\Delta\theta^2 \\ &+ \frac{2}{3} K_z \sin 2\theta_H \,\Delta\theta^3 \end{aligned} . \quad \text{(S2)}$$

The last term in Eq. (S2) represents the higher-order potential that generates the nonlinear diode voltage. Under this potential, an increase in the magnetization-precession angle induces a change in the precession center. If the coefficient 2/3 $K_z \sin 2\theta_H$ is positive, the precession center θ_m decreases from its initial value θ_H. On the contrary, if it is negative, θ_m increases. In this experiment, the in-plane magnetic anisotropy occurs because K_z is negative at a bias current of −1.39 mA. Here, a dc bias current of −1.39 mA in the MTJ corresponds to a bias voltage of −347 mV at a θ_H of 90°. Therefore, θ_m increases in the ranges $0° < \theta_H < 90°$ and $180° < \theta_H < 270°$, and it decreases in the ranges $90° < \theta_H < 180°$ and $270° < \theta_H < 360°$, as shown in Figs. S2(c) and S2(d), respectively. Because the pinned-layer magnetization is in the −*y* direction, increasing the precession angle

decreases the resistance in the range $-90° < \theta_H < 90°$, and increases it in the range $90° < \theta_H < 270°$. Because the nonlinear diode voltage V_{NLD} is the product of the resistance change and dc bias current, a positive nonlinear diode voltage is generated in the range $-90° < \theta_H < 90°$, and a negative voltage is generated in the range $90° < \theta_H < 270°$, as shown in Fig. S2(e). Our discussion based on Fig. S2(e) is consistent with the experimental result shown in Fig. S2(b).

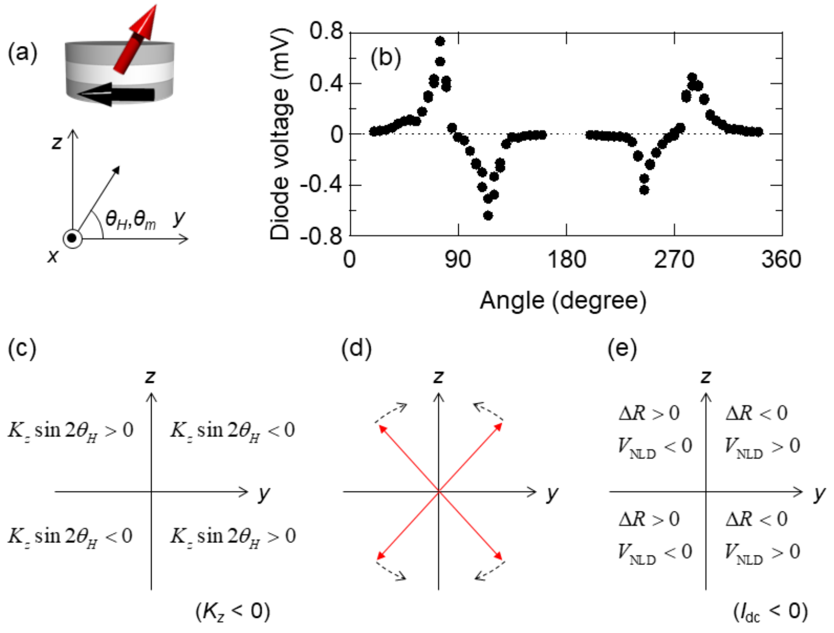

Supplementary Figure S2.(a) Schematic of free (red) and pinned (black) layer magnetization in the magnetic tunnel junction. The elevation angles of magnetic field and magnetization are defined as θ_H and θ_m, respectively. (b) Dependence of diode voltage on elevation angle of magnetic field. (c) Sign of the coefficient of higher-order potential along *y*–*z* plane under negative perpendicular magnetic anisotropy. (d) Red arrows represent free-layer magnetization. Dashed black arrows indicate the change in magnetization direction due to magnetization precession under a higher-order potential. (e) Sign of resistance change ΔR and nonlinear diode voltage V_{NLD} along *y*–*z* plane under negative dc bias current.

Supplementary Note 4: Effect of spin-torques on responsivity

We discuss the effect of various spin-torques, such as HCMA, VCMA, and spin-transfer torque, on the responsivity. Using the HCMA value calculated from Fig 6(b), the oscillation amplitude of the magnetic anisotropy field $\frac{2\Delta K_{\mathrm{HCMA}}}{M_{\mathrm{s}}} = \frac{4V_{\mathrm{dc}}V_{\mathrm{ac}}}{M_{\mathrm{s}}SR}HCMA$ can be evaluated as 500 μT at a microwave power of −55 dBm with an insertion loss of 1.16 dB. Here, ΔK_{HCMA} is the oscillation amplitude of magnetic anisotropy; M_{s} is the saturation magnetization; V_{dc} and V_{ac} are the dc and ac voltage, respectively; and S and R are the area and resistance of the MTJ, respectively. In the same manner, the linear VCMA value can be calculated using k_1, the coefficient of the linear term in the expansion of the perpendicular magnetic anisotropy (Fig 6(b)). The oscillation amplitude of the magnetic anisotropy field $\frac{2\Delta K_{\mathrm{VCMA}}}{M_{\mathrm{s}}} = \frac{2V_{\mathrm{ac}}}{M_{\mathrm{s}}t_{\mathrm{FeB}}t_{\mathrm{MgO}}}VCMA$ can be calculated to be 21.5 μT under the same conditions.

Spin-transfer torque can be characterized by the amplitude of the diode voltage under a perpendicular magnetic field because the anisotropy change does not affect the magnetization dynamics in this condition. Figure S3(a) shows the diode spectra of an MTJ of diameter of 130 nm under various perpendicular magnetic fields at the microwave power of −25 dBm without bias voltage. These

results are fitted by the function

$$V_{diode} = A\frac{\Delta f \cdot f^2}{\left(f^2 - f_r^{\,2}\right)^2 + \left(\Delta f \cdot f\right)}. \qquad \text{(S1)}$$

Here, f, f_{r}, and Δf represent the frequency of the microwave, the resonant frequency, and the full width at half maximum of the resonant peaks, respectively; A is the amplitude of the peak. Figure S3(b) shows the magnetic field dependence of the amplitude A. By solving Landau-Lifshitz-Gilbert-Slonczewski equation, the amplitude is calculated to be $A = \frac{gI_{\mathrm{ac}}}{4\pi G_0}\gamma B_{\mathrm{stt}}$, where I_{ac} is ac current, γ is gyro-magnetic ratio, and B_{stt} is the effective field of spin-transfer torque. The quantities g and G_0 are $\frac{G_{\mathrm{P}} - G_{\mathrm{AP}}}{G_{\mathrm{P}} + G_{\mathrm{AP}}}$ and $\frac{G_{\mathrm{P}} + G_{\mathrm{AP}}}{2}$, respectively, where G_{P} and G_{AP} are the parallel and anti-parallel conductance, respectively. We used the amplitude at 100 mT because, as shown in Fig S1(c), the free-layer magnetization is almost saturated at that field intensity, and a larger magnetic field increases the modulation of the pinned-layer magnetization. Using the amplitude of 3.4 μVGHz at the 100 mT, we obtained the effective magnetic field of spin-transfer torque B_{stt} = 308 μT at a microwave power of −25 dBm, including an insertion loss of 1.16 dB. This value can be converted to B_{stt} = 9.8 μT at a microwave power of −55 dBm, the condition of the experiment shown in

Fig 2(b). Therefore, the spin-torque due to HCMA is dominant in this experiment.

Although we suggested that large HCMA (large rf-spin-torque) produces large responsivity, it is not obvious under the mechanism in this manuscript. Thus, here, we discuss the relation between rf-spin-torque and responsivity induced by HCMA. From the power dependence of responsivity as shown in Fig 3, larger spin-torques induce higher responsivity. The diode voltage is proportional to the microwave power, and heat induced spin-torque in dc-biased MTJ is proportional to the microwave voltage, implying that the diode voltage is proportional to the square of rf spin-torque. In our system, because the HCMA is high and exerts a large spin-torque, the diode voltage increases, and a high responsivity is obtained.

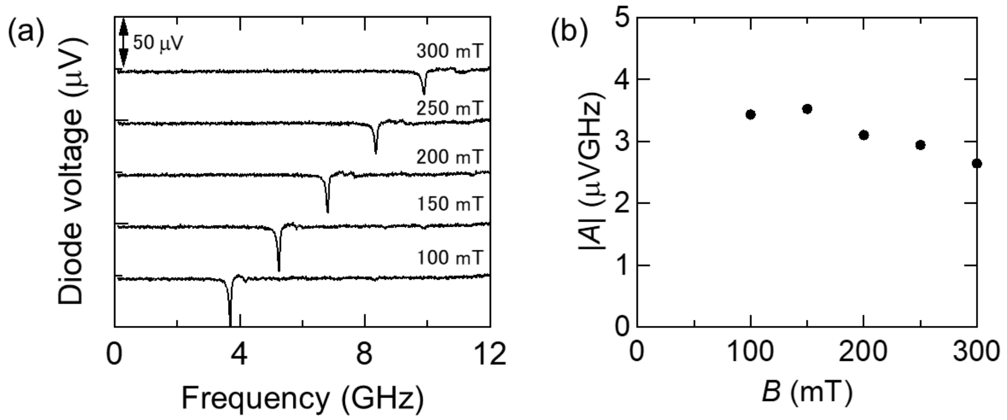

Supplementary Figure S3. (a) Frequency dependence of diode voltage under various perpendicular magnetic fields. (b) Perpendicular magnetic field dependence of peak intensity *A* obtained by the fitting.

Supplementary Note 5: Frequency tunability and dynamic range of responsivity

As shown in Fig 2(b), the diode voltage is obtained at 0.59 GHz with a line width of only 0.1 GHz. This frequency is tunable by magnetic-field and bias-voltage conditions. Figure S4 shows the diode spectra at various magnetic field conditions. The peak frequency can be modulated from 0.59 GHz to 0.74 GHz; there is a tradeoff between increasing peak frequency and decreasing responsivity.

As shown in Fig. 3, the dynamic range of the diode voltage is approximately from 0.1 mV to 10 mV. To expand this dynamic range, improvement of the signal to noise ratio (SNR) is necessary. In this experiment, the diode voltage reaches a noise equivalent voltage (NEV) of 0.1 mV at the microwave power of 0.1 nW. The decrease in the NEV enhances the dynamic range. This SNR can be improved by increasing the ferromagnetic-layer thickness. Spin-transfer torque and VCMA are inversely related to the FeB thickness. HCMA also diminishes with thickness, but the decrease is smaller than that in spin transfer torque or VCMA. This is because the increase in temperature of the ferromagnetic layer is affected by the MgO layer through which the heat

flows. Therefore, HCMA is preferable for improving the dynamic range of the responsivity.

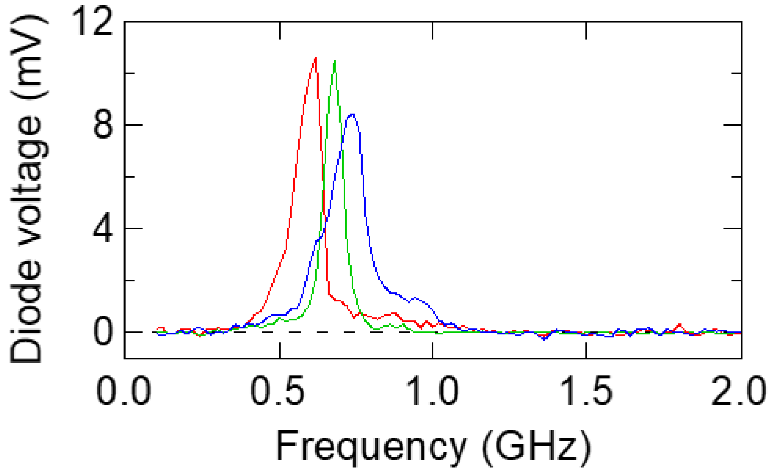

Supplementary Figure S4 Frequency dependence of diode voltage at various magnetic field conditions (*B, θ, φ*). Red, green, and blue curves represent the diode spectra at the magnetic field conditions (50 mT, 79°, 45°), (54 mT, 78°, 50°), and (60 mT, 80°, 50°), respectively.

Supplementary Note 6: Optimal conditions for obtaining noise equivalent power

We explain the results of the noise equivalent power (NEP) measurements considering the spin-torque diode effect. We searched for the optimal conditions for NEP in the magnetic field range of 44 mT – 60 mT, azimuthal angles between 6° – 12°, and rotation angles in the range 20° – 60°. Figure S5 shows the frequency dependence of the diode voltage. We obtained a minimum NEP of $2.4\times10^{-12}\ \mathrm{W}/\sqrt{\mathrm{Hz}}$ under the optimal conditions of B = 54 mT, θ = 12°, and φ = 50° at a dc bias current of −2.6 mA and an input microwave power of −55 dBm including the insertion loss of 1.16 dB. Here, the NEP is described by noise ($\mathrm{V}/\sqrt{\mathrm{Hz}}$) / responsivity (V/W). The noise associated with the diode voltage is characterized by the standard deviation of the voltage in the range of 1.3 – 2.0 GHz with a time constant of 100 ms. The responsivity is (3.33 ± 0.01)×10^6 V/W under these conditions. Taking into consideration the insertion loss of 1.16 dB, the responsivity is (4.35 ± 0.01)×10^6 V/W.

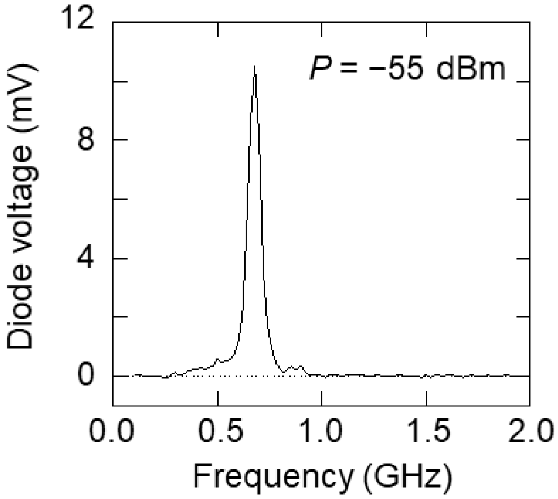

Supplementary Figure S5. Frequency dependence of diode voltage under optimal conditions for noise equivalent power: magnetic field intensity B = 54 mT, azimuthal angle θ = 12°, and rotational angle φ = 50° at a dc bias current of −2.6 mA. The input microwave power P is −55 dBm.

Supplementary References

1 Miwa, S. *et al.* Highly sensitive nanoscale spin-torque diode. *Nat. Mater.* **13**, 50-56, doi:10.1038/nmat3778 (2014).

2 Kiselev, S. I. *et al.* Microwave oscillations of a nanomagnet driven by a spin-polarized current. *Nature* **425**, 380-383 (2003).

3 Goto, M. *et al.* Microwave amplification in a magnetic tunnel junction induced by heat-to-spin conversion at the nanoscale. *Nat. Nanotechnol.* **14**, 40-43, doi:10.1038/s41565-018-0306-9 (2019).

4 Zhang, L. *et al.* Ultrahigh detection sensitivity exceeding 10^5 V/W in spin-torque diode. *Appl. Phys. Lett.* **113**, doi:10.1063/1.5047547 (2018).
